# Spiritual Leadership in Hotels and Service Performance Under Emotional Demands: The Mediating Role of Emotion Regulation Self-Efficacy

**DOI:** 10.3390/bs16060888

**Published:** 2026-06-01

**Authors:** JaeWon Shin, HyoungChul Shin

**Affiliations:** 1 Department of Global Tourism Management, Seokyeong University, Seoul 02713, Republic of Korea; marketgarden38@skuniv.ac.kr; 2 Department of Foodservice and Culinary Management, Kyonggi University, Seoul 03746, Republic of Korea

**Keywords:** spiritual leadership, emotion regulation self-efficacy, service performance, hotel industry

## Abstract

This study analyzes the relationships among spiritual leadership, emotion regulation self-efficacy, and service performance in the hotel industry where emotional labor is emphasized. Data were collected through an online survey of hotel employees at three-star or higher-grade hotels in Korea. A total of 347 valid samples were analyzed using confirmatory factor analysis and structural equation modeling. Four hypotheses were established. First, spiritual leadership was expected to positively relate to emotion regulation self-efficacy. Second, emotion regulation self-efficacy would be positively related to service performance. Third, spiritual leadership was hypothesized to have a positive relationship with service performance. Fourth, emotion regulation self-efficacy was expected to mediate a positive relationship between spiritual leadership and service performance. The results of the analysis supported all four hypotheses. The findings indicate that spiritual leadership enhances employees’ emotion regulation self-efficacy, improving emotion regulation and, in turn, service performance. Therefore, hotel organizations should consider improving service performance and competitiveness by developing leadership strategies and educational programs that strengthen employees’ emotion regulation capabilities.

## 1. Introduction

The hotel industry is a representative service sector characterized by frequent customer contact and strong labor-intensive features. Employees’ roles in the service provision process are a key determinant of organizational performance. In particular, hotel employees must perform high levels of emotional labor due to continuous interaction with customers. In this environment, not only employee behavior but also psychological resources significantly influence service quality, customer loyalty, and organizational reputation (Ribeiro et al., 2020; Lee & Ok, 2015). Therefore, hotel companies must consider not only employees’ job performance but also their psychological stability and internal motivation to maintain competitiveness.

In this context, prior hospitality research has emphasized leadership as a key factor shaping employees’ positive attitudes and behaviors (Ma et al., 2024; Bani-Melhem et al., 2021). Leaders go beyond establishing and directing management strategies; they play a central role in shaping organizational culture and influencing employees’ thoughts and behaviors (George et al., 1999). Moreover, in an increasingly uncertain management environment, the influence of leadership on employee behavior and role performance continues to expand (Ahearne et al., 2005; Chen & Yang, 2012; DiLiello & Houghton, 2006).

However, as organizational structures have become more horizontal and employees’ autonomy has increased in recent years, traditional authoritative and performance-oriented leadership styles are no longer sufficient to satisfy employees’ intrinsic motivations and psychological needs (Cardoso & de Andrade Baptista, 2015). Criticism has been raised that such leadership approaches tend to focus primarily on achieving organizational goals while neglecting employees’ emotional and psychological well-being (McLaughlin, 2005). As an alternative, spiritual leadership—which emphasizes the pursuit of meaning and internal motivation—has gained increasing attention.

Spiritual leadership is defined as leadership that fosters internal motivation and promotes psychological well-being through shared vision, hopes/beliefs, and altruistic love (L. W. Fry, 2003). It is characterized by fostering organizational commitment and voluntary participation by enabling employees to perceive their work as meaningful rather than merely task-oriented (Moxley, 2000). Previous studies have also reported that spiritual leadership positively influences various organizational performance variables, including job satisfaction, organizational commitment, and organizational citizenship behavior (Jiang et al., 2023; Göçen & Şen, 2021; Kumar, 2017).

In emotionally demanding environments such as the hotel industry, employees’ ability to manage their emotions is a crucial determinant of service performance. Self-efficacy refers to an individual’s belief in their ability to perform tasks successfully and an important psychological resource for predicting service behavior and job performance (Abuelhassan & AlGassim, 2022; Etehadi & Karatepe, 2019; Karatepe et al., 2007). Emotion regulation self-efficacy refers to the belief in an individual’s ability to recognize and control emotions (Dacre Pool & Qualter, 2012; Kirk et al., 2008). It is defined as confidence in managing emotions during job performance (Galla & Wood, 2012) and plays a crucial role in emotional labor contexts.

Emotion regulation self-efficacy is a subjective evaluation of one’s ability to maintain positive emotions and control negative ones, and it ensures smooth interactions with customers in service settings (Bandura et al., 2003; Caprara et al., 2008).

In the hotel industry, the ability to control negative emotions arising from customer interactions is directly related to service quality. Therefore, emotion regulation self-efficacy is an important variable in explaining service performance. Furthermore, emotion regulation self-efficacy can be influenced by individual characteristics and organizational environmental factors, such as leadership behavior, which plays an important role in the formation of these psychological resources (Gumah et al., 2021; Ashfaq et al., 2021). Leaders who understand and support employees’ emotions can strengthen emotion regulation self-efficacy by relieving employees’ negative emotions and providing psychological stability. In this respect, spiritual leadership likely serves as an important antecedent by addressing employees’ emotional needs and providing psychological support.

Nevertheless, prior research has primarily focused on the direct effects of spiritual leadership on attitudinal outcomes, with limited attention to how leadership translates into actual service performance through psychological resources. Although emotional labor requires continuous emotion regulation, previous studies have not sufficiently explained the role of emotion-related self-efficacy in linking leadership to service performance.

To address this gap, this study examines how spiritual leadership influences service performance by focusing on emotion regulation self-efficacy as a key intervening factor. Unlike general self-efficacy studied in broader management contexts, this research specifically targets ‘emotion regulation self-efficacy’ to reflect the high-intensity emotional demands of the hotel industry. Building on the Conservation of Resources (COR) theory (Hobfoll, 1989), we argue that the continuous emotional labor required in hotel settings acts as a significant drain on employees’ psychological energy. In this context, spiritual leadership serves as an essential contextual resource that initiates a ‘resource gain spiral’ (Xanthopoulou et al., 2007), providing employees with the spiritual and emotional support needed to replenish their internal stores.

This process prevents the depletion of emotional resources caused by frequent interactions with customers and enhances the belief in one’s ability to manage stress. As managing negative emotions from customer interactions is a primary job demand in the hospitality sector (Salehzadeh et al., 2015), spiritual leadership plays a vital role in fostering emotional resilience and maintaining service consistency. Specifically, by offering a vision and altruistic love, spiritual leaders strengthen members’ confidence in regulating their emotions even under high pressure (Huang, 2022). By doing so, this study contributes to the literature by providing a more detailed explanation of how leadership affects service performance in emotional labor settings and highlights the unique role of spiritual values as a source of emotional resilience. Unlike previous studies that focus on general self-efficacy, this research identifies ‘emotion regulation self-efficacy’ as a critical personal resource for hotel employees facing high emotional demands. By integrating Conservation of Resources (COR) theory (Hobfoll, 1989), we elucidate the mechanism through which spiritual leadership triggers a ‘resource gain spiral’, ultimately enhancing service performance. This approach provides a more nuanced theoretical grounding for explaining the psychological recovery and performance of service providers.

## 2. Theoretical Background

### 2.1. Spiritual Leadership

Leadership is a key factor in shaping the attitudes and behaviors of an organization’s members and in determining its performance. It remains a central research topic in the fields of organizational behavior and human resource management (Yukl & Michel, 2006). Traditional leadership research has largely focused on management methods emphasizing economic rewards and performance, centering on the exchange relationships between supervisors and subordinates (Hughes et al., 2006). However, recent leadership research emphasizes immaterial factors such as members’ values, sense of meaning, and intrinsic motivation (Chen & Li, 2013). Amid these developments, workplace spirituality has gained attention as a concept reflecting members’ sense of meaning and internal motivation. Workplace spirituality refers to an organizational environment in which members recognize purpose and meaning in their work and develop mutual understanding and empathy through interpersonal relationships (Milliman et al., 1999; Wagner-Marsh & Conley, 1999). It can also be understood as a cultural framework that fosters joy and compassion in the organization and encourages positive employee interactions (Giacalone & Jurkiewicz, 2003). This concept provides the theoretical basis for spiritual leadership.

Spiritual leadership is defined as the values, attitudes, and behaviors of a leader that promote members’ intrinsic motivation and psychological well-being and was conceptualized by L. W. Fry (2003). Fry described spiritual leadership as a process through which leaders and members motivate themselves and others based on a shared sense of calling and community (L. W. Fry, 2003). This leadership process promotes voluntary participation and commitment by allowing members to perceive their work as meaningful rather than merely task-oriented (Moxley, 2000). Furthermore, spiritual leadership extends beyond the instrumental application of positive attitudes; it is fundamentally rooted in a robust anthropology that recognizes the spiritual dimension as an essential and inherent part of the human person (Elkins et al., 1988). While it is often discussed in relation to performance, its core purpose is to address the fundamental human need for transcendence and purpose, facilitating a transformative process for both the leader and the follower.

Spiritual leadership comprises three key components: vision, hope/faith, and altruistic love (L. W. Fry, 2003). Vision provides direction by presenting organizational goals and shared values (Becker, 2007). Hope/faith fosters the belief that challenges encountered in pursuing organizational goals can be overcome (MacArthur, 1998). Altruistic love promotes positive organizational relationships and alleviates negative emotions through mutual respect, care, and consideration among members (L. W. Fry, 2003). Through these components, spiritual leadership serves as a process of holistic human formation, where work becomes a venue for individuals to realize their full potential and internal growth as human beings, rather than being treated merely as a means to achieve organizational ends.

Previous studies show that spiritual leadership produces positive organizational outcomes. For example, it has been reported to enhance organizational performance by improving employee commitment and innovation capabilities (M. Wang et al., 2019) and to positively influence emotional commitment and the formation of organizational trust (Jeon & Choi, 2020). It also contributes to employees’ psychological stability and job satisfaction (Gündüz, 2017). Thus, spiritual leadership is an important leadership approach that fosters positive behavior and performance by strengthening members’ internal motivation and psychological resources. Its importance is particularly evident in the hotel industry, where emotional labor is high, as it provides a meaningful framework that sustains the human spirit amidst demanding service interactions.

### 2.2. Emotion Regulation Self-Efficacy

Emotion regulation self-efficacy refers to an individual’s belief in their ability to recognize and regulate emotions (Bandura et al., 2003; Caprara et al., 2008) and extends the concept of self-efficacy within social cognitive theory. Self-efficacy refers to the belief that an individual can successfully perform a particular task (Bandura, 2006), and influences behavioral choices, levels of effort, and adaptation to the environment. From this perspective, emotion regulation self-efficacy serves as a key indicator of an individual’s ability to manage emotions and self-regulate. It is distinct from general self-efficacy in that it refers specifically to beliefs about emotional functioning (Bandura et al., 2003). It is defined as the belief in one’s ability to maintain positive emotions and control negative ones (Alessandri et al., 2014) and is closely related to the regulation of emotional experiences and expressions (Gross, 2008). It influences individuals’ emotional responses and behavior extending beyond actual regulatory ability to include expectations about one’s capacity to control emotions (Kirk et al., 2008; Caprara et al., 2006; Kohli & Jaworski, 1990).

Previous studies demonstrate that emotion regulation self-efficacy plays a critical role in psychological stability and adaptation. Positive emotion regulation self-efficacy improves life satisfaction and enhances responses to challenges (Bandura, 1997; Bryant et al., 2005). Conversely, negative emotion regulation self-efficacy alleviates negative thinking and emotional distress, thereby reducing psychological maladjustment (Kirk et al., 2008; Caprara et al., 2006). It also functions as a psychological resource that helps individuals manage job-related stress and supports appropriate emotional expression and regulation in interpersonal contexts (Schaubroeck & Merritt, 1997).

Particularly, in emotionally demanding environments that emphasize emotional labor, such as the hotel industry, the ability to effectively manage negative emotions, conflicts, and time pressure during customer interactions significantly influences service performance. In this context, emotion regulation self-efficacy is a key factor in maintaining consistent behavior and enhancing customer satisfaction.

In this study, emotion regulation self-efficacy is conceptualized in two dimensions: regulation of negative emotions and regulation in situations involving distress and frustration. This dimensional focus reflects the fact that hotel employees primarily experience emotional burdens related to negative emotions and stressful situations. Accordingly, the ability to control negative emotions and recover quickly from difficult situations is considered more directly relevant to service performance than the expression of positive emotions.

Thus, this study analyzes emotion regulation self-efficacy in situations involving negative emotions, distress, and frustration. The theoretical positioning of this variable is grounded in the Conservation of Resources (COR) theory (Hobfoll, 1989), which suggests that individuals strive to obtain, retain, and protect their resources. In the hotel industry, constant emotional labor acts as a ‘resource drain’ (Salehzadeh et al., 2015). Emotion regulation self-efficacy, in this sense, is not just a psychological state but a ‘key resource’ that allows employees to invest their remaining energy into delivering high-quality service. By identifying spiritual leadership as an antecedent that fosters this specific personal resource (Xanthopoulou et al., 2007), this study moves beyond the traditional ‘leadership–performance’ link and offers a more nuanced mechanism tailored to service-oriented environments (Huang, 2022).

### 2.3. Service Performance

Service performance is defined as employee behaviors and outcomes at customer contact points that contribute to achieving the goals of a service organization (Churchill et al., 1993). In the service industry, employees who interact directly with customers play a key role in determining service quality and organizational performance, and their ability to adapt to various service situations and respond effectively to customer needs is critical (De Jong et al., 2004). At the individual level, service performance encompasses the behaviors and attitudes employees exhibit during customer interactions, including the ability to meet customer expectations and elicit satisfaction (Liao & Chuang, 2004). Furthermore, service performance extends beyond simple task execution to encompass customer response processes, problem-solving abilities, and the consistent delivery of service (Miller, 2013).

Previous research has shown that service performance is an important outcome variable at both the organizational and individual levels. At the organizational level, service performance improves in environments that emphasize service orientation (Kohli & Jaworski, 1990). At the individual level, employees’ attitudes and behaviors significantly influence service performance (Hartline & Ferrell, 1996). In particular, employee behavior at customer contact points is closely related to customers’ perceived service quality, which directly affects the company’s competitiveness (Brady & Cronin, 2001; Lam et al., 2015). In addition, organizations use employee service performance as a key indicator of both overall performance and individual evaluation (Kelley, 1992), highlighting its importance in securing competitive advantage.

Thus, service performance represents a behavioral outcome of employee–customer interactions and is particularly important in the hotel industry, where emotional labor is highly emphasized. In such contexts, employees’ emotional management abilities and psychological resources are expected to play a crucial role in determining service performance under the various emotional demands that arise in the customer response process.

### 2.4. Correlation Among Variables

Empirical studies directly examining the relationship between spiritual leadership and emotion regulation self-efficacy remain limited. Therefore, this study explains this relationship by examining the link between leadership and employees’ emotional and psychological resources. According to the Conservation of Resources (COR) theory (Hobfoll, 1989), individuals strive to obtain and protect valuable resources to cope with stress. In this context, spiritual leadership acts as a critical contextual resource that facilitates a ‘resource gain spiral’ (Xanthopoulou et al., 2007), providing employees with the psychological energy needed to manage emotional demands.

Spiritual leadership promotes internal motivation and psychological well-being, strengthens commitment to the organization, and plays an important role in satisfying members’ psychological needs (Reave, 2005). In addition, a spiritually enriched workplace environment contributes to psychological stability, encourages voluntary work participation, and clarifies workers’ perception of organizational goals (Duchon & Plowman, 2005; Rego & Pina e Cunha, 2008). In this context, leaders who understand and support members’ emotional and psychological states can positively affect emotional stability and self-regulation. For example, transformational leadership has been found to improve emotion regulation self-efficacy, and reduce job burnout (Cui, 2023), while spiritual leadership has also been reported to significantly influence members’ self-efficacy (Junaidi, 2026). These findings suggest that value-based leadership behaviors serve as important antecedents of members’ psychological resources. Therefore, spiritual leadership is expected to positively affect members’ confidence in regulating their emotions, and the following hypothesis is proposed.

**Hypothesis** **1.** *Spiritual leadership has a positive relationship with emotion regulation self-efficacy.*

Bandura (1982) explained that emotional support and positive feedback from leaders can improve members’ self-efficacy. Employees with high self-efficacy exhibit more active behavior and higher levels of job performance compared to those with lower self-efficacy. From this perspective, self-efficacy is a key psychological factor influencing an individual’s behavioral choices, effort, and performance. In particular, emotion regulation self-efficacy refers to the belief that individuals can effectively manage and control their emotions in emotionally demanding situations. In the hospitality industry, where constant customer interaction leads to ‘resource drain’ (Salehzadeh et al., 2015), this specific efficacy becomes a ‘key resource’ that allows employees to invest their remaining energy into high-quality service performance. Previous studies have shown that emotion regulation self-efficacy improves job satisfaction and positively affects the performance of employees working in service roles (Osman, 2016; Bordin et al., 2006). Moreover, employees with high emotion regulation self-efficacy are more likely to maintain emotional stability and exhibit positive service attitudes during customer interactions, leading to customer satisfaction and improved service quality (Hallowell et al., 1996). Thus, emotion regulation self-efficacy is a key psychological resource for maintaining consistent service behavior in emotional labor environments. Therefore, emotion regulation self-efficacy is expected to be an important prerequisite for improving service performance, and the following hypothesis is proposed.

**Hypothesis** **2.** *Emotion regulation self-efficacy has a positive relationship with service performance.*

Leadership is an important factor in determining customer service performance at both the individual and organizational levels in service organizations (Q. Wang, 2019). In particular, spiritual leadership has been shown to positively affect service behavior by reinforcing members’ internal motives and organizational commitment. Previous studies indicate that spiritual leadership promotes employees’ active service behavior and reinforces customer-oriented attitudes within the organization’s culture (Jeon & Choi, 2021; Yang et al., 2021). It also aligns organizational goals with individual values, enhances the meaning of work, and strengthens members’ sense of voluntary efforts and responsibilities (Bayighomog & Araslı, 2019). These effects contribute to improved service behavior and, ultimately, enhanced service performance. In addition, spiritual leadership improves both organizational and individual performance by fostering trust, a sense of belonging, and a productive work environment (L. W. Fry, 2003; L. Fry & Kriger, 2009). This relationship suggests that spiritual leadership can directly drive excellence in service by creating a resourceful organizational climate that supports employees’ spiritual and emotional needs. Therefore, spiritual leadership is expected to be an important prerequisite for improving service performance, and the following hypothesis is proposed.

**Hypothesis** **3.** *Spiritual leadership has a positive relationship with service performance.*

Spiritual leadership also plays an important role in developing emotional resources by strengthening members’ psychological stability and internal motivation. As noted, it is a prerequisite for improving emotion regulation self-efficacy, which directly affects behavior and performance in service situations.

Based on this logic, emotion regulation self-efficacy is expected to mediate the relationship between spiritual leadership and service performance. This mediation mechanism is theoretically grounded in the ‘resource gain spiral’ of the Conservation of Resources (COR) theory (Hobfoll, 1989; Xanthopoulou et al., 2007). In this process, the contextual resources provided by spiritual leaders—such as vision, hope/faith, and altruistic love—help employees build and expand their personal resources, specifically emotion regulation self-efficacy. These accumulated psychological resources are then converted into positive organizational outcomes, such as enhanced service performance. Furthermore, this framework provides a robust explanation for the partial mediation observed in this study. According to COR theory, spiritual leadership not only fosters personal resources but also creates a resourceful work environment that can directly drive performance independently of individual efficacy beliefs (Huang, 2022). This multifaceted influence explains the persistence of the direct effect often found in value-based leadership studies.

**Hypothesis** **4.** *Emotion Regulation Self-Efficacy mediates the positive relationship between spiritual leadership and service performance.*

**Figure 1:**
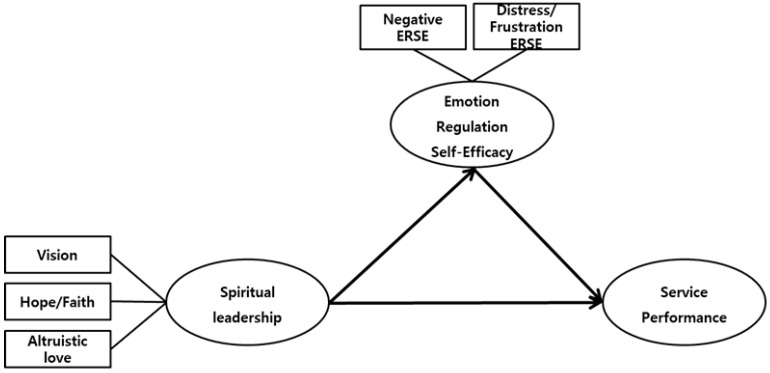
Study model.

This study examines the relationships among spiritual leadership, emotion regulation self-efficacy (ERSE), and service performance, as shown in Figure 1. (ERSE = Emotion Regulation Self-Efficacy).

## 3. Materials & Methods

### 3.1. Data Collection and Method

This study surveyed employees working in three-star or higher hotels in Korea from 1 December to 31 December 2025. The hotel industry, a representative service sector characterized by frequent customer contact and high levels of emotional labor, is well-suited to examining the influence of leadership and emotional resources. In particular, hotels with three-star or higher ratings provide standardized structures and service at a certain level or higher, making them suitable for systematically analyzing members’ behaviors and psychological responses. Judgment sampling was used to select respondents who met the research objectives, considering industry and job characteristics. Non-probability sampling was appropriate given the practical constraints associated with probability sampling in this context.

Data were collected from hotel employees through a single-source online survey, where participants provided self-reported responses for all variables, including perceived spiritual leadership, emotion regulation self-efficacy, and service performance. A questionnaire was developed using Google Forms, and the survey link was distributed through hotel partners. Collaborators explained the purpose and procedures of the study to the respondents and obtained their informed consent. All responses were voluntary, and it was stated in advance that study participants would be provided with anonymity and confidentiality. It was also clearly stated that the collected data would be used solely for research purposes. Ethical standards were observed by sufficiently explaining to the respondents their rights and the purpose of the study. A total of 351 responses were collected. After excluding four responses due to the same response pattern and unfaithful responses, 347 valid responses were used for the final analysis.

Data analysis was performed using IBM SPSS 30.0 and AMOS 30.0 following Anderson and Gerbing’s (1988) two-step approach, first testing the measurement model and then analyzing the structural model. Confirmatory factor analysis (CFA) was conducted to verify the validity and reliability of the measurement model. Convergent validity and internal consistency were confirmed, and discriminant validity among constructs was reviewed. Discriminant validity was confirmed when the mean variance extraction value (AVE) for each constituent concept was greater than the square value of the correlation coefficient. Subsequently, structural equation modeling (SEM) was used to evaluate model fit and to test the causal relationships among latent variables. Hypothesis verification was conducted using structural model analysis, and a bootstrapping procedure was applied to assess the mediating effect. Confidence intervals for indirect effects were calculated to determine statistical significance.

### 3.2. Measurement

The questionnaire was developed based on previous studies on spiritual leadership, emotional regulation, self-efficacy, and service performance, with items adapted to suit the research context. All questions were measured using a five-point Likert scale (1 = not at all, 5 = very much).

Spiritual leadership was measured based on L. W. Fry’s (2003) framework, drawing on subsequent studies (M. Wang et al., 2019; Jeon & Choi, 2020). It consists of three dimensions: vision, hope/faith, and altruistic love, measured with a total of nine items representing each dimension. Example questions include: “The leader of our organization presents a convincing vision for the future” and “Our leader humanely respects and cares for our members.” Emotion regulation self-efficacy was adapted from prior studies in service contexts, particularly the scale developed by Caprara et al. (2008). Given that the hotel industry frequently involves negative emotions and stressful customer interactions, this study focused on two dimensions: negative emotion regulation self-efficacy and regulation in distress and frustration situations. The dimension related to positive emotional expression was excluded, as the study focuses on aspects more directly related to actual performance ability in emotional labor situations. It consisted of six questions, including statements such as “You can effectively control your emotions even if you are angry” and “You can overcome frustration even if things don’t go well.” Service performance was measured based on previous studies (Liao & Chuang, 2004; Babakus et al., 2009), focusing on performance in emotionally demanding service situations. It was assessed with four items, including “handling customer complaints calmly and effectively” and “maintaining consistent service quality under pressure.”

## 4. Results

### 4.1. Demographics of the Participants

Table 1 presents the demographic characteristics of the sample. The sample consisted of 67.4% male and 32.6% female participants. In terms of age, the largest group was participants in their 20 s (40.1%). Regarding education level, the majority held an associate degree (59.9%). Most respondents were permanent employees (81.3%).

### 4.2. Reliability and Validity

#### 4.2.1. Confirmatory Factor Analysis

CFA was conducted to assess the fit of the measurement model, and the results are presented in Table 2. In this study, spiritual leadership and emotion regulation self-efficacy were conceptualized as higher-order constructs. To simplify the analysis and improve model fit, an index integration approach using the average of each lower dimension was applied as the primary factor. This parceling approach was employed to maintain a parsimonious model and improve the stability of parameter estimates, especially given the complex higher-order nature of the constructs. By reducing the total number of estimated parameters, this method enhances the model’s degrees of freedom and provides more reliable results for testing structural relationships in relatively complex models.

The results supported the adequacy of the proposed three-factor model (i.e., spiritual leadership, emotion regulation self-efficacy, and service performance). The overall model fit indices indicated an acceptable fit to the data (χ^2^ = 37.989, df = 17, *p* < 0.001; χ^2^/df = 2.235; RMR = 0.016; GFI = 0.974; AGFI = 0.944; NFI = 0.979; IFI = 0.988; TLI = 0.980; CFI = 0.988; RMSEA = 0.060). All standardized factor loadings exceeded the recommended threshold of 0.50 and were statistically significant. Composite reliability values exceeded 0.70, indicating satisfactory internal consistency (Sarstedt et al., 2021). These results confirm adequate convergent validity and reliability of the measurement model.

#### 4.2.2. Discriminant Validity

Convergent and discriminant validity were assessed to evaluate the adequacy of the measurement model. Discriminant validity was examined using the Fornell–Larcker criterion, which compares the average variance extracted (AVE) of each construct with squared correlations (Fornell & Larcker, 1981). Discriminant validity is established when the AVE of each construct exceeds its squared correlations with other constructs. As shown in Table 3, the highest correlation was observed between spiritual leadership and emotion regulation self-efficacy (r = 0.741), with a squared correlation of 0.549. The AVE values for spiritual leadership (0.827) and emotion regulation self-efficacy (0.791) both exceeded this value. In addition, the AVE for service performance (0.718) was greater than its squared correlations with other constructs. These results indicate that discriminant validity is adequately established for all constructs.

#### 4.2.3. Common Method Bias

To assess potential common method bias (CMB), Harman’s single-factor test was conducted. The first unrotated factor accounted for 49.01% of the total variance, slightly below the commonly accepted threshold of 50%, although relatively close to the cut-off. These results suggest that common method bias may not severely threaten the validity of the findings; however, caution is still warranted due to the single-source design (Podsakoff et al., 2003). Additionally, a full collinearity test was performed. All variance inflation factor (VIF) values were below the conservative threshold of 3.3, providing further evidence that common method bias is unlikely to materially distort the observed relationships (Kock, 2015).

### 4.3. Hypothesis Testing

SEM was conducted using AMOS 30.0 to test the proposed hypotheses. The overall model fit indices indicated an acceptable fit (χ^2^ = 37.989, df = 17, *p* < 0.001; χ^2^/df = 2.235; RMR = 0.016; GFI = 0.974; AGFI = 0.944; NFI = 0.979; IFI = 0.988; TLI = 0.980; CFI = 0.988; RMSEA = 0.060), satisfying recommended thresholds (Sarstedt et al., 2021). The results of the structural model are presented in Table 4. First, spiritual leadership had a significant positive effect on emotion regulation self-efficacy (β = 0.741, t = 12.062, *p* < 0.001), supporting Hypothesis 1. Second, emotion regulation self-efficacy had a significant positive effect on service performance (β = 0.492, t = 5.161, *p* < 0.001), supporting Hypothesis 2. Third, spiritual leadership had a significant positive effect on service performance (β = 0.197, t = 2.232, *p* < 0.05), supporting Hypothesis 3. To examine the mediating effect, a bootstrapping procedure was employed. The indirect effect of spiritual leadership on service performance through emotion regulation self-efficacy was significant (β = 0.364, *p* = 0.004). Since both the direct and indirect effects were significant, emotion regulation self-efficacy was found to partially mediate the relationship between spiritual leadership and service performance. Therefore, Hypothesis 4 was supported.

## 5. Discussion and Conclusions

This study empirically examined the relationships among spiritual leadership, emotion regulation self-efficacy, and service performance among hotel employees and analyzed the mediating role of emotion regulation self-efficacy. It is particularly significant in providing a comprehensive analysis of the structural relationships among leadership, employees’ psychological resources, and service performance in the hotel industry, where emotional labor is emphasized. Regarding the age distribution, the majority of respondents (77%) were young adults in their 20 s and 30 s. Given that emotional self-regulation skills often develop naturally through the aging process, the high concentration of younger participants in this study suggests that spiritual leadership may play an even more critical role in fostering these psychological resources during the earlier stages of professional life. The main findings are as follows.

First, spiritual leadership was found to have a positive relationship with emotion regulation self-efficacy. This indicates that spiritual leadership plays an important role in strengthening members’ belief in their ability to regulate emotions by satisfying psychological needs, inducing internal motivation, and providing psychological stability. In particular, spiritual leadership allows members to recognize meaning and purpose within the organization through factors such as vision presentation, hope and belief, and altruistic love, which can help alleviate negative emotions that arise in emotional labor situations. These results support the results of previous studies (Reave, 2005; Duchon & Plowman, 2005; Rego & Pina e Cunha, 2008; Cui, 2023). Moreover, this study confirms that spiritual leadership influences not only attitudinal variables but also psychological resources such as emotion regulation self-efficacy.

Second, emotion regulation self-efficacy was positively related to service performance. This positive relationship suggests that employees’ belief in their ability to manage the emotionally demanding hotel environment translates into improved service behavior and performance. Employees with high emotion regulation self-efficacy are more likely to maintain emotional stability during customer interactions and exhibit consistent service attitudes even under stress, thereby enhancing service quality. These results support previous studies (Osman, 2016; Bordin et al., 2006; Snipes et al., 2005; Wong & Law, 2017) and reaffirm the importance of emotional self-regulation ability in service performance within emotional labor environments.

Third, spiritual leadership was found to be positively related to service performance, implying that the leader’s vision, hope and faith, and consideration for employees reinforce their internal motivation and commitment, leading to improved service behavior and performance. In particular, spiritual leadership can foster responsibility and voluntary participation by instilling a sense of belonging and mission, thereby improving service quality. These results align with the findings of previous studies (Jeon & Choi, 2021; Yang et al., 2021; Bayighomog & Araslı, 2019; L. Fry & Kriger, 2009).

Fourth, emotion regulation self-efficacy partially mediated the relationship between spiritual leadership and service performance. This partial mediation implies that while spiritual leadership enhances service performance by building employees’ emotional resources, it also exerts a direct influence through other motivational and social mechanisms. Theoretically, according to the Conservation of Resources (COR) theory, spiritual leadership acts as a contextual resource that fosters a ‘resource gain spiral,’ where increased self-efficacy becomes a personal resource that directly translates into higher performance. The persistence of a significant direct effect suggests that spiritual leadership may also improve performance by aligning individual values with organizational goals or by fostering a sense of calling, independent of the emotional regulation process.

### 5.1. Theoretical Implications

This study provides the following theoretical implications. The theoretical significance of this study lies in its direct application of Conservation of Resources (COR) theory to the specific context of spiritual leadership and emotion regulation in the hotel industry. Unlike previous studies that focused on general psychological resources, this research specifically identifies ‘emotion regulation self-efficacy’ as a potent personal resource that mediates the effect of spiritual leadership on performance.

First, spiritual leadership reinforces hotel employees’ self-efficacy, enabling effective emotional control in challenging or stressful situations (Huang, 2022). Existing studies have primarily focused on attitude variables, such as job satisfaction and organizational commitment. However, this study shows that spiritual leadership also shapes psychological competencies, including emotional regulation and self-efficacy. This finding indicates that spiritual leadership extends beyond conveying of values to supporting emotional control in emotional labor contexts.

Second, it was confirmed that technical competence, emotional stability, and self-efficacy are important factors in hotel employees’ service performance. While existing service performance studies emphasized functional factors such as job performance ability and service skills, the results of this study show that service performance is substantially influenced by employees’ emotional states and psychological resources. In particular, the joint contribution of spiritual leadership and emotion regulation self-efficacy to service performance empirically suggests that leadership affects performance indirectly through employees’ psychological states (Supriyanto et al., 2020). The finding of partial mediation provides a deeper understanding of the complex path between leadership and performance. It indicates that emotion regulation self-efficacy is a critical, but not the sole, mechanism. Alternative mechanisms, such as increased organizational identification or the fulfillment of a ‘sense of membership’—core components of spiritual leadership—may account for the remaining direct effect. By confirming this partial mediation, this study suggests that spiritual leadership drives service excellence through both the strengthening of specific psychological competencies and the broader cultivation of a meaningful work environment.

Third, this study deviates from existing performance-based leadership research by emphasizing the importance of spiritual leadership’s inner meaning, values, and community within spiritual leadership. In particular, it suggests that leadership fostering members’ psychological stability and emotional balance can work more effectively in environments with high emotional labor, such as the hotel industry, than in contexts that directly require performance. These results underscore the importance of emotional and psychological dimensions often overlooked in leadership research and support the view that spiritual leadership plays a practical role in emotional labor contexts.

Furthermore, while this study confirms the linear path from leadership to performance, it highlights the importance of considering boundary conditions that may influence these relationships. According to the Job Demands–Resources (JD–R) model (Xanthopoulou et al., 2007), the impact of spiritual leadership may vary depending on the ‘emotional labor intensity’ of the specific hotel role. In environments where customer interactions are exceptionally demanding, the spiritual support provided by leaders becomes an even more critical buffer against resource depletion. By recognizing these contextual variations, this study provides a more sophisticated theoretical foundation for understanding how spiritual leadership interacts with the complex emotional landscape of the hospitality industry.

### 5.2. Practical Implications

This study offers practical implications for strengthening human resource management and service competitiveness in the hotel industry.

First, organizations should implement education and environments that reinforce spiritual leadership. Hotel managers must stimulate employees’ intrinsic motivation by demonstrating leadership that gives their jobs meaning and purpose beyond the simple role of management at the level of work instruction. Particularly, sharing the organization’s vision and values with members continuously helps employees recognize their work as delivering value to customers (Meng, 2016; M. Wang et al., 2019), encouraging them to act as active creators of service experiences, and not just role-play as performers.

Second, the emotional competencies of hotel employees should be developed systematically. Practical education and training programs, such as emotional labor management workshops and stress management training, are needed to effectively address the emotional situations that arise in the customer response process. In addition, operating psychological support systems—including mentoring and coaching based on vision presentation, belief, and altruistic consideration—which are key elements of spiritual leadership, can help employees regulate their emotions and strengthen job confidence (Haver et al., 2013). These programs should be implemented regularly and integrated into the organizational training systems to ensure continuous development of emotional regulation capabilities.

Third, it is necessary to reorganize the criteria for evaluating hotel employees’ service performance. Performance evaluation should be more multifaceted. While the hotel industry often relies on quantitative indicators, such as sales and room share, emotional stability and customer satisfaction should also be included as important performance factors. Managers should regularly analyze customer feedback to assess how employees’ emotional regulation and service attitudes shape the customer experience (Ashtar et al., 2021). These factors should be incorporated into formal evaluation criteria and supported by systematic use of customer feedback data.

Fourth, it is necessary to develop an organizational culture grounded in spiritual leadership that fosters psychological stability. This strategic approach requires creating environments where employees can express emotions and experiences relatively freely and share problem situations. Efforts to encourage the formation of relationships based on mutual respect and consideration and trust among members are essential (Yasin et al., 2023). Organizations should establish formal mechanisms (e.g., regular feedback sessions and peer support systems) to ensure that employees can share emotional experiences and receive support.

### 5.3. Limitations and Future Research

This study has several limitations that suggest directions for future research.

First, the analysis relies on cross-sectional data collected at a single point in time. Therefore, there is a limit to fully interpreting the causal relationship between variables. Future studies should verify the relationship between variables over time by applying a longitudinal design.

Second, as all variables were measured using self-reported scales from a single source at a single point in time, the potential for common method bias (CMB) cannot be entirely ruled out. Although Harman’s single-factor test showed that the first factor accounted for 49.01% of the total variance, which is below the commonly accepted 50% threshold, this value is relatively close to the cut-off. Therefore, common method bias may not severely threaten the validity of the findings. However, caution is still warranted due to the single-source, self-reported design. To further assess this issue, a full collinearity VIF test was conducted, and all VIF values were below 3.3, providing additional evidence that common method bias is unlikely to materially distort the observed relationships. However, future research should adopt a longitudinal design or collect multi-source data to more rigorously control for potential common method bias.

Additionally, this study did not include specific control variables, such as employee age, tenure, or hotel grade. In particular, as literature suggests that emotional self-regulation tends to improve with age (Carstensen et al., 1999; Urry & Gross, 2010), it is important to consider whether the self-reported efficacy was influenced by demographic functions rather than leadership alone. Future studies should include these demographic and organizational factors as control variables to isolate the unique impact of spiritual leadership. Specifically, testing these relationships by comparing responses across different age groups would help isolate the developmental effects of aging from the influence of spiritual leadership, thereby enhancing the validity of the findings.

Third, emotion regulation self-efficacy was examined as a parameter, but other relevant psychological and organizational factors that could explain the relationship between leadership and performance were not fully considered. Specifically, future research should incorporate potential moderators such as ‘service climate’ or ‘psychological safety’ to enhance the model’s explanatory depth. A positive service climate may amplify the effect of spiritual leadership by providing an organizational foundation for spiritual values to thrive. Similarly, when psychological safety is high, employees may feel more secure in utilizing their emotion regulation self-efficacy to address customer needs without fear of negative repercussions. Examining these boundary conditions will provide more practical and sophisticated insights into how different hotel environments influence the effectiveness of spiritual leadership.

Fourth, emotion regulation self-efficacy was measured primarily in terms of negative emotions and situational control. While appropriate to emotional labor contexts, this approach does not capture positive emotional expression and maintenance. Future studies should adopt more comprehensive measures to consider the various subdimensions of emotion regulation self-efficacy.

### 5.4. Conclusions

In conclusion, this study applies Conservation of Resources (COR) theory to explain how spiritual leadership relates to hotel employees’ service performance under emotionally demanding conditions. The findings suggest that spiritual leadership, characterized by vision, hope, and altruistic love, positively contributes to employees’ emotion regulation self-efficacy, which in turn enhances service performance. These results highlight the importance of psychological and emotional resources in maintaining service quality in hospitality settings. As emotional demands continue to increase in the hospitality industry, organizations may benefit from leadership approaches that support employees’ emotional stability and internal motivation.

## Figures and Tables

**Table 1:** Demographic characteristics of the participants.

Demographic Factors	Category	Number of Participants	Percentage (%)
Gender	Male	234	67.4
	Female	113	32.6
Age	20 s	139	40.1
	30 s	128	36.9
	40 s	66	19
	50 years and older	14	4
Education	High school diploma or less	32	9.2
	Associate degree	208	59.9
	Bachelor’s degree (4-year university)	91	26.2
	Graduate degree or higher	16	4.6
Working period	Less than 5 years old	153	44.1
	Between 5 and 10 years	80	23.1
	Between 10 and 15 years	53	15.3
	Between 15 and 20 years	41	11.8
	More than 20 years	20	5.8
Employment type	Permanent employee	282	81.3
	Non-regular employee	65	18.7
Total		347	100

**Table 2:** Confirmatory factor analysis.

Factor and Variable		Standardized Loading	S.E	C.R	AVE	Composite Construct Reliability (CCR)	Cronbach’s α
Spiritual Leadership	Vision	0.843	-	-	0.883	0.958	0.895
	Hope/Faith	0.88	0.042	23.324 ***			
	Altruistic love	0.843	0.042	24.149 ***			
Emotion Regulation Self-Efficacy	Negative ERSE	0.861	-	-	0.881	0.937	0.834
	Distress/Frustration ERSE	0.831	0.05	19.784 ***			
Service Performance	SP1	0.811	-	-	0.746	0.921	0.897
	SP2	0.824	0.044	20.819 ***			
	SP3	0.837	0.04	21.248 ***			
	SP4	0.852	0.042	21.740 ***			

χ^2^ = 80.177 (df = 24, *p* = 0.000), CMIN/DF = 3.341, RMR = 0.016, GFI = 0.968, AGFI = 0.940, NFI = 0.974, IFI = 0.982, TLI = 0.973, CFI = 0.982, RMSEA = 0.068. *** *p* < 0.001.

**Table 3:** Discriminant validity.

Factor	Spiritual Leadership	Emotion Regulation Self-Efficacy	Service Performance
Spiritual leadership	0.827 ^(1)^	0.549 ^(3)^	0.315
Emotion regulation Self-Efficacy	0.741 ^(2)^	0.791	0.407
Service performance	0.562	0.638	0.718

The notation used in the analysis is as follows: (1) Diagonal represents the Average Variance Extracted (AVE); (2) The area below the diagonal represents the correlation coefficient for the constructs (r); (3) The area above the diagonal represents the square of the correlation coefficient (r^2^).

**Table 4:** Results of the structural equation model analysis.

		Estimate	*t*-Value	*p*-Value	Indirect Effect		Decision
	Process (Hypothesis)				Estimate	*p*	
H1	Spiritual leadership → Emotion regulation self-efficacy	0.741	12.062 ***	0			Supported
H2	Emotion regulation self-efficacy → Service performance	0.492	5.161 ***	0			Supported
H3	Spiritual leadership → Service performance	0.197	2.232 **	0.026			Supported
H4	Spiritual Leadership → Service Performance (mediating effects of Emotion Regulation Self-Efficacy)	0.197	2.232 **	0.026	0.364 **	0.004	Supported

** *p* < 0.01; *** *p* < 0.001.

## Data Availability

The data presented in this study are available on request to the corresponding author.
